# Enhanced quantitative urine culture technique, a slight modification, in detecting under-diagnosed pediatric urinary tract infection

**DOI:** 10.1186/s13104-019-4875-y

**Published:** 2020-01-03

**Authors:** Januka Thapaliya, Priyatam Khadka, Shovana Thapa, Chenu Gongal

**Affiliations:** 1 Tri-Chandra Multiple Campus, Tribhuvan University, Ghantaghar, Kathmandu, Nepal; 2 International Friendship Children’s Hospital, Kathmandu, Nepal

**Keywords:** UTI, EQUC, Uropathogens, Children, ESBL, MDR, XDR

## Abstract

**Objectives:**

The pediatric urinary tract infection (UTI) often remains under-diagnosed or neglected owing to non-specific clinical presentations, patients failing to describe the actual situation and of clinical practice in diagnosis. The study was aimed to determine the etiologies of UTI in children with enhanced quantitative urine culture (EQUC) technique.

**Results:**

Of enrolled 570 pediatric urine samples, the significant growth positivity was higher in EQUC 92 (16.15%) compared to standard urine culture (SUC) 73 (12.80%) technique. 20.6% of the significant isolates as detected with EQUC were missed on the SUC technique. The age group, in range 1–4 years, was more prone to the infection, where *E. coli* was the commonest pathogen. EQUC detected, probably all isolates, contributing UTI i.e. multidrug-resistant (MDR), extensive drug-resistant (XDR), and extended-spectrum β-lactamase (ESBL) producers, as some of them skipped on the SUC technique. Of total organisms isolated from EQUC, 46% were ESBL producer, 56.5% were MDR, and 1.4% were XDR. However, 40.5% ESBL, 44% MDR but no XDR detected on SUC. Hence a simple modification on conventional culture protocol could be a crucial modification for the detection of etiologies, contributing UTI, and hence to reduce inapt antimicrobial burden.

## Introduction

Urinary tract infection is one of the most common infections with a leading cause of morbidity and mortality in children [1]. However, in this age-group, the infection often remains under-diagnosed or neglected owing to non-specific clinical presentations, patients failing to describe the actual situation, and of conventional clinical practice of diagnosis [2]. Since the 1950s, the clinical practice has relied upon SUC protocol as a gold standard in detecting etiologies contributing UTI; nevertheless, continues to be questioned for its precision in both clinical diagnosis and implicated antimicrobial therapy [3]. Hence, the precise diagnostic protocol is mandatory to reduce the superfluous antimicrobial burden and to truncate the possible adverse consequences, in the pediatric population [1–3].

Although, the documented incidence of the infection ranges from 23.1 to 37.4% in the Nepalese population [4]. In Nepal, and most developing countries, the pediatric UTIs cases are treated empirically due to lacking appropriate diagnostic protocol, unavailability of standard therapeutic guidelines, and undocumented resistivity trend of the pathogens in local and regional levels [4–6]. Therefore, a precise diagnosis of the etiologies and its resistivity status against the preferred antibiotics is crucial for successful clinical management and prophylaxis. With these backdrops, we conducted a study to determine the etiology of UTI among children with EQUC, a slight modification on the SUC technique, to trace the significant etiologies missing on SUC.

## Main text

### Materials and methods

#### Study design and sample population

The cross-sectional study was carried out from April 2017–October 2017 in International Children Friendship Hospital, Kathmandu, Nepal. The study hospital is a tertiary referral center for children. The totals of 570 urine samples were enrolled in our study. The study populations were infants and children, not exceeding 14 years old, seeking treatment for presumed UTI.

#### Inclusion and exclusion criteria

Children enrolled in the pediatric outpatient department or admitted in ward, with clinical diagnosis as UTI, were included. The clinical diagnosis was made by the corresponding unit pediatrician relying upon the patient presenting with fever and/or with symptoms suggestive to UTI.

The urine samples which grew more than one type of organism were considered as a contaminant (in those children who had previously known the history of antimicrobial therapy within 48 h before attending the hospital) and hence, excluded from the study.

#### Sample collection and analysis

The urine samples (collected either with urethral catheterization, or supra-pubic aspirations and pediatric urine collection bag for toilet-untrained children, and mid-stream urine for toilet-trained children) were processed semi-quantitatively with both EQUC and SUC techniques.

In brief, the SUC protocol used 1 μl of urine, spread quantitatively onto 5% sheep blood [blood agar plate (BAP)] and MacConkey agars (BD BBL Prepared Plated Media; Hi-Media) and incubated aerobically at 35 °C for 24 h. The urine samples were then inoculated the corresponding subset of EQUC conditions using three urine volumes (1 μl, 10 μl, and 100 μl) and additional plating conditions. Each urine sample was spread quantitatively onto (BAP, chocolate Agar,); chocolate agar plates were incubated in 5% CO_2_ at 35 °C for 48 h; BAP and MacConkey agars were incubated aerobically at 35 °C for 48 h. Only the confluent growth of a single organism, with a count of ≥ 10^5^colony forming units (CFU)/ml were presumed as significant growth. For EQUC, the significant colony was calculated about volume inoculated as described by Brincat et al. with a little modification [3]. Further, microbial identification was done by using the recommended in-house set of biochemical tests and phenotypic characteristics.

#### Antimicrobial susceptibility testing

The antimicrobial susceptibility of bacterial isolates against different antibiotics was tested by the disk diffusion method [modified Kirby-Bauer method] on Mueller–Hinton agar (Hi-Media, India) following standard procedures recommended by the Clinical and Laboratory Standards Institute (CLSI), Wayne, PA, USA [7]. The antimicrobials used were: penicillin [ampicillin (10 μg)], penicillins with β-lactamase inhibitors [ampicillin–sulbactam (10/10 μg), amoxicillin–clavulanic acid (10 μg)], narrow-spectrum cephalosporin [cefazolin (30 μg)], extended-spectrum cephalosporins [ceftazidime (30 μg), ceftriaxone (30 μg), cefepime (30 μg)], cephamycin [cefoxitin (30 μg)], anti-pseudomonal penicillins with β-lactamase inhibitors [piperacillin–tazobactam (100/10 μg)], monobactam [aztreonam (30 μg)], carbapenems [imipenem (10 μg), meropenem (10 μg)], aminoglycosides [gentamicin (10 μg), amikacin (30 μg)], fluoroquinolones [ciprofloxacin (5 μg), ofloxacin (5 μg)], folate pathway inhibitor [co-trimoxazole (25 μg)], and polymyxin [colistin (10 μg)]. The interpretations of antibiotic susceptibility results were made according to the zone size interpretative standards of CLSI [7].

#### Identification of MDR, XDR and potential ESBL

MDR and XDR isolates were identified about the combined guidelines of the European Centre for Disease Prevention and Control (ECDC) and the Centers for Disease Control and Prevention (CDC) [8]. The isolate resistant to at least one antimicrobial from three different groups of first-line drugs tested was regarded as MDR; while those resistant to at least one agent in all but two or fewer antimicrobial categories (i.e., bacterial isolates remains susceptible to only one or two categories) are termed as XDR [7, 8]. For the confirmation, of all potential ESBL producers, the Combined Disk test (CDT), as recommended by CLSI was performed in all isolates [7].

#### Data management and statistical analysis

Data obtained (patient’s demographics and the results) were entered and managed on Microsoft Excel (version 2010 Microsoft Corporation, USA); the relation of variables was calculated in frequencies and percentages.

### Result

#### Patients’ demographics

Of 570 pediatric urine samples, the significant bacterial growth detected: 92 (16.14%) with EQUC and 73 (12.8%) with SUC protocol.

Of 92 UTI cases, the infection was higher in female children 67 (21.1%) compared to males 25 (9.96%). The age group, in range 1–4 years 33 (42.9%), and the patient admitted in wards 39 (18.1%) were more prone to the infection (Table 1).

**Table 1:** Patients’ demographics

Patients demographics	Uropathogen detected (%)	Uropathogen not detected (%)	Total
*Gender*			
Male	25 (9.96)	226 (90.04)	251
Female	67 (21.1)	252 (78.9)	319
*Age group*			
< 1 year	15 (9.3)	146 (90.7)	161
1 to 4 years	33 (42.9)	44 (57.1)	77
5 to 9 years	29 (20)	116 (80)	145
10 to 14 years	15 (8.1)	172 (91.9)	187
*Patients distribution*			
Out-patient	53 (14.98)	301 (85.02)	354
In-patient	39 (18.1)	177 (81.9)	216

#### EQUC vs. SUC in uropathogens detection

EQUC detected all possible etiologies, contributing UTIs, in the clinically suspected subjects; as reported: “no growth” with the standard urine culture protocol. Of total enrolled cases, 92 significant UTIs cases were detected with EQUC; however, only 73 with SUC technique. 20.6% of isolates are being missed with the SUC technique. A statistical outline was drawn with paired t-test (Additional file 1: Table S1(a) (b)).

**Fig. 1:**
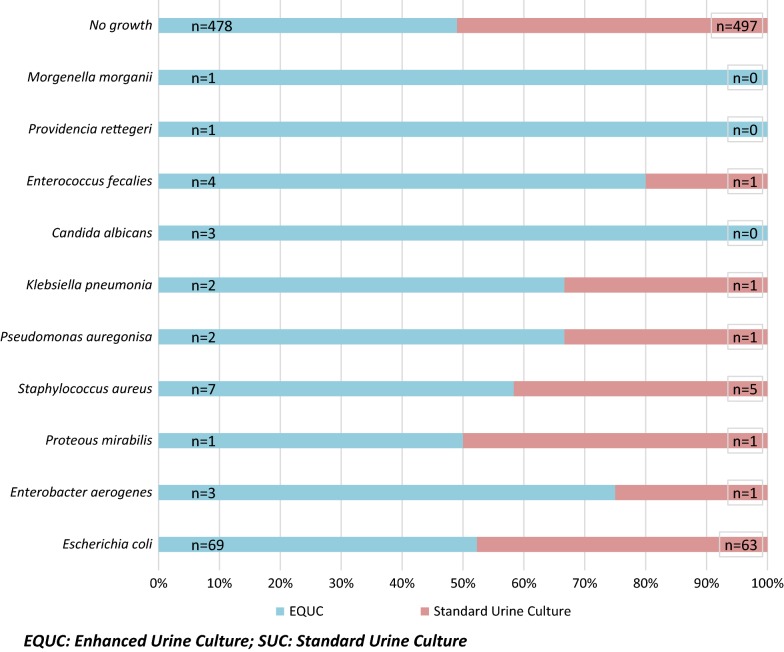
Uropathogens isolated with EQUC and SUC technique

Among the study population, *E. coli* predominantly found as culprits preceding UTIs: 69 (75%) with EQUC and 63 (68.4%) with the SUC technique. The uropathogens i.e. *Candida albicans, Provedencia retegerii,* and *Morganella morganii* failed to grow on SUC technique; although, they grew on EQUC (Fig. 1).

#### Resistivity pattern of uropathogens

Most *E. coli* isolates were resistant to ampicillin (77%), followed by ciprofloxacin (65.07%), cotrimoxazole (51%), nitrofurantoin (33.3%), gentamycin (25.3%), cefixime (22.2%) and ceftriaxone (22.2%). Nevertheless, the entire strains revealed high susceptibility (up to 100%) with colistin and tigecycline (Additional file 2: Table S2).

#### MDR, XDR, and ESBL producers

Of the total 69 *E. coli* isolates subjected for antimicrobial susceptibility testing: ESBL 32 (46%); MDR 39 (56.5%) and XDR 1 (1.4%) detected with EQUC. The SUC protocol, however, detected ESBL 28 (40.57%), MDR 31 (44%) and XDR (nil) (Table 2).

**Table 2:** Uropathogens detected as ESBL, MDR, and XDR with EQUC and SUC technique

Organism isolated	Growth positivity (%)	ESBL (%)	MDR (%)	XDR (%)
EQUC	92 (16.15)	32 (46.0)	39 (56.5)	1 (1.4%)
Standard	73 (12.80)	28 (40.57)	31 (44.0)	0
Difference	19 (3.35)	4 (5.5)	8 (12.6)	1 (1.4%)

### Discussion

The pediatric UTI, cases are often under-diagnosed or neglected due to non-specific clinical presentations and of clinical practice—relying upon in vitro culture report; however, it may incur dire consequences. Therefore, a precise diagnosis is crucial for clinical management. In this perspective, our study underscores the insufficiencies in SUC protocol in detecting significant etiologies, possibly MDR and XDR isolates, and advocates for a slight modification concerning the sample volume being inoculated.

Among the study population, the incidence of urinary tract infection was 16.14%; and *E. coli* (68.5%) was the commonest pathogen. The analogous rates have been reported earlier from neighboring hospitals [4–6] and studies from other nations [9, 10]. Alongside, significantly more females up to 72.0% had UTI substantiating with other similar studies [5, 6]. In our study, the children of the age group 1-4 years were more prone to the infection. Our premise is comparable to findings conducted in a nearby hospital where less than 6 years were high-risk age categories [4, 6]. The immune status, sanitation, and ascending infection with fecal flora possibly are the reasons behind such upshots in this age group.

The EQUC technique, a simple but effective technique, was embraced to determine etiologies in the clinically UTI suspected children. The same technique was applied to the women experiencing UTI like symptoms, before [3]. EQUC detected all possible etiologies, contributing UTIs as reported: “no growth” with the standard urine culture protocol. Of total 92 detected cases of UTI, 73 were isolated with SUC—conceding 20.6% being missed. However, the study population was different i.e. clinically suspected women, but similar finding favoring EQUC over SUC was attained.

Among 69 *E. coli* isolates, the highest resistance (77% each) was attributed to ampicillin followed by ciprofloxacin (65.07%). The resistance pattern was similar as observed by Parajuli et al. (87%) to ampicillin and (78%) to ciprofloxacin. Likewise, our findings are coherent, regarding resistance trend of the isolate against ampicillin and ciprofloxacin, to that of Ansari et al. (74%) and (77%); the age-group subjects was different, however [11]. The isolate, *E. coli*, found resistant to cefixime (22.2%) and ceftriaxone (22.2%). Among antimicrobials tested, colistin (100%), imipenem (nearly 99%) were sensitive. Therefore, a second and third-generation cephalosporin (cefixime and ceftriaxone) could be choices; polymyxin (colistin) and carbapenem (imipenem) could better be opted-in treating childhood UTI.

The etiology, *Staphylococcus aureus,* in pediatric UTI is commonly associated with acquired infection preceding from in-dwelling catheters or other devices [12]. Of 7 isolates of *Staphylococcus aureus*, 5 were recovered from the patient after catheterization; 2 of the isolates were resistant to ampicillin and cotrimoxazole; while one each found resistant to ofloxacin, cloxacilline, cefoxitine, cephalexine, and nitrofurantoin. The single isolate was methicillin-resistant *Staphylococcus aureus* (MRSA); as reported by some authors in the pediatric population [13, 14].

The uropathogens (*Candida albicans, Provedencia retegerii, and Morganella morganii*) were isolated with EQUC while missed on SUC; although, these pathogens were cited, as the significant etiologies contributing childhood UTI [15–18]. Hence from our study, it can be clinched that each uropathogens, possibly significant causative agent, may have its’ own unique threshold bacterial load, concerning the volume to be inoculated on culture media.

Apart from these, our study underscores 5.5% of ESBL, 12.6% MDR, and 1.4% of XDR isolates were about to be missed if only SUC has opted. In this study, MDR and XDR isolates were found 56.5% and 1.4% respectively while 46% of uropathogens were found ESBL producers. Nevertheless, an increasing pattern of resistance trend in uropathogens, along with MDR rates has been reported, among pediatric isolates, from Nepal [5, 6, 19]. The level of drug-resistant uropathogens among the children in this study is of serious concern; nevertheless, the exact figures with exact anti-microbial resistance status (that possibly missed on SUC) were not analyzed before.

In most developing countries like Nepal, the higher antimicrobial burden preceding inapt therapeutic guidelines among pediatric patients might be attributable to the intimidating scenario [4–6]. Existing higher rates of ESBL, MDR, and XDR; necessitates the use of carbapenem, colistin, tigecycline, and other mono-antimicrobial therapies (cephamycins, fosfomycin and nitrofurantoin); however, the resistance to these potent therapeutic options may not be stood robust till longer due to emerging MDR strains [11, 20–23].

#### Conclusion

EQUC detects uropathogens, possibly MDR, XDR, and ESBL producers, which could be reported: “no growth” on the SUC protocol. Therefore, a simple modification, on conventional culture protocol could be a crucial modification for the detection of etiologies, and to reduce inapt antimicrobial burden.

## Limitation of the study

We could not encompass the large sample size with this new modification—the EQUC technique. Our study was restricted to phenotypic anti-microbial resistance detection excluding the identification of different beta-lactamases producing isolates. Although, genomic sequencing provides insightful resistance trend due to constricted laboratory resources was not included in our study.

## Supplementary information

**Additional file 1: Table S1.** (a) Paired sample t-test EQUC and SUC technique. (b) Paired differences.

**Additional file 2: Table S2.** Resistivity pattern of uropathogens.

## Data Availability

Data generated or analyzed during this study are included in this published article and remaining are available from the corresponding author on reasonable request.

## Abbreviations

ASM: American Society for Microbiology
ATCC: American Type Culture Collection
CLSI: Clinical and Laboratory Standard Institute
*E. coli*: *Escherichia coli*
EQUC: enhanced quantitative urine culture
ESBL: extended-spectrum beta-lactamase
MDR: multiple drug-resistant
SUC: standard urine culture
UTI: urinary tract infection
XDR: extensive drug-resistant
