# Supplementary material for: Enhanced quantitative urine culture technique, a slight modification, in detecting under-diagnosed pediatric urinary tract infection
Source: BMC Res Notes. 2020 Jan 3;13:5. doi: 10.1186/s13104-019-4875-y (PMC6942300; doi:10.1186/s13104-019-4875-y)
Supplement: Supplementary file 1 — Additional file 1: Table S1. (a) Paired sample t-test EQUC and SUC technique. (b) Paired differences. [file 13104_2019_4875_MOESM1_ESM.docx]

| Additional File 1  Table S1  (a) Paired samples statistics | | | | |
| --- | --- | --- | --- | --- |
| Pair 1 | mean | N | Std. deviation | Std. Error mean |
| Uropathogen detected with SUC | 1.19 | 570 | 0.677 | 0.028 |
| Uropathogen detected with EQUC | 1.3386 | 570 | 1.18 | 0.049 |

| (b)Paired Differences | | | | | | | |
| --- | --- | --- | --- | --- | --- | --- | --- |
| Pair 1 | Mean | S.D | S.E Mean | 95% C I Difference | t | d f | Sig. (2 tailed) |
|  |  |  |  | Lower |  |  |  |
| Uropathogen (Standard)- (EQUC) | -.15088 | .99650 | .04174 | -.23286 | -3.615 | 569 | .001 |
