# Supplementary material for: Enhanced quantitative urine culture technique, a slight modification, in detecting under-diagnosed pediatric urinary tract infection
Source: BMC Res Notes. 2020 Jan 3;13:5. doi: 10.1186/s13104-019-4875-y (PMC6942300; doi:10.1186/s13104-019-4875-y)
Supplement: Supplementary file 2 — Additional file 2: Table S2. Resistivity pattern of uropathogens. [file 13104_2019_4875_MOESM2_ESM.docx]

| Additional file 2(Table S2): Resistivity pattern of uropathogen | | | |
| --- | --- | --- | --- |
| Organism | Antibiotics used | Susceptibility pattern | |
|  |  | Sensitive (%) | Resistant (%) |
| *Escherichia coli* | Ampicillin | 18(26.1%) | 51(73.90 %) |
|  | Cotrimoxazole | 32(43.4%) | 37(53.60%) |
|  | Nitrofurantoin | 47(66.%) | 22(33.30%) |
|  | Ciprofloxacin | 25(37%) | 44(63.0%) |
|  | Gentamycin | 52(75.4%) | 17(25.60%) |
|  | Cefexime | 55(79.8%) | 14(20.20%) |
|  | Ceftriaxone | 53(76.8%) | 16(23.20%) |
|  | Colistin | 100% | 0 |
|  | Tigecycline | 100% | 0 |
|  | Imipenum | 68(98.5%) | 1(1.5%) |
| *Klebsiella pneumoniae* | Ampicillin | 0 | 2(100%) |
|  | Cotrimoxazole | 1(50%) | 1(50%) |
|  | Nitrofurantoin | 2(100%) | 0 |
|  | Ciprofloxacin | 0 | 2(100%) |
|  | Gentamycin | 1(50%) | 1(50%) |
|  | Cefexime | 1(50%) | 1(50%) |
|  | Ceftriaxone | 1(50%) | 1(50%) |
| *Enterobacter aerogenes* | Ampicillin | 1(33%) | 2(67%) |
|  | Cotrimoxazole | 2(67%) | 1(33%) |
|  | Nitrofurantoin | 3(100%) | 0 |
|  | Ciprofloxacin | 2(67%) | 1(33%) |
|  | Gentamycin | 3(100%) | 0 |
|  | Cefexime | 1(33%) | 2(67%) |
|  | Ceftriaxone | 1(33%) | 2(67%) |
| *Pseudomonas aerugonisa* | Pipracillin/Tazobactam | 2(100%) | 0 |
|  | Ceftazidium | 2(100%) | 0 |
|  | Levofloxacin | 2(100%) | 0 |
|  | Amikacin | 1(50%) | 1(50%) |
|  | Ciprofloxacin | 2(100%) | 0 |
|  | Nitrofurantoin | 2(100%) | 0 |
| *Enterococcus faecalis* | Ampicillin | 1(25%) | 3(75%) |
|  | Ofloxacin | 3(75%) | 1(25%) |
|  | Nitrofurantoin | 4(100%) | 0 |
|  | Vancomycin | 4(100%) | 0 |
|  | Teicoplanin | 4(100%) | 0 |
|  | Amoyclave | 4(100%) | 0 |
|  | Cefrtiaxone | 1(25%) | 3(75%) |
|  | Amikacin | 3(75%) | 1(25%) |
| *Staphylococcus aureus* | Ampicillin | 4(57.14%) | 3(42.86%) |
|  | Ofloxacin | 6(85.7%) | 1(14.3%) |
|  | Cotrimoxazole | 5(71.4%) | 2(28.6%) |
|  | Cloxacillin | 5(71.4%) | 2(28.6%) |
|  | Cefoxitine | 5(71.4%) | 2(28.6%) |
|  | Cephalexin | 5(71.4%) | 2(28.6%) |
|  | Nitrofurantoin | 6(85.7%) | 1(14.3%) |
| The antimicrobial susceptibility test was not performed for *Candida albicans* | | |  |
